# The incidence and risk factors of contralateral knee arthroplasty after primary unilateral unicompartmental knee arthroplasty

**DOI:** 10.1097/MD.0000000000026825

**Published:** 2021-08-13

**Authors:** Xiaodan Huang, Hua Li, Baicheng Chen, Decheng Shao, Haiyun Niu, Jianchao Wang, Guang Yang

**Affiliations:** The Third Department of Joint Orthopedics, The Third Hospital of Hebei Medical University, Hebei, China.

**Keywords:** incidence, retrospective study, risk factors, unicompartmental arthroplasty

## Abstract

Unicompartmental knee arthroplasty (UKA) is one of the commonly used surgical methods for unicompartmental osteoarthritis in recent years. Although the prognosis of the operated knee has been widely studied, there are relatively little data on the natural history of the contralateral knee after unilateral replacement. The aim of this study was to explore the incidence and risk factors of consequential knee arthroplasty in patients with bilateral knee osteoarthritis (KOA) after receiving primary unilateral UKA, so as to provide a theoretical basis for making a more comprehensive treatment strategy for patients with KOA.

We conducted a retrospective study and enrolled patients with bilateral KOA received unilateral UKA from June 2015 to December 2019 in the third department of joint orthopedics, the third hospital of Hebei Medical University. The patients were divided into replacement group and non-replacement group according to whether the contralateral knee joint received knee arthroplasty. Information about treatment of contralateral knee joint was collected from medical records to determine the incidence. Univariate analysis and multivariate logistic regression analysis were performed to identify the independent risk factors.

A total of 502 patients were enrolled in this study. The incidence of contralateral knee arthroplasty was 38.64%. In the univariate analysis, vertical angle of mechanical axis, knee joint's internal and external joint space, Kellgren–Lawrence (K-L) classification, femoral tibial angle were the significant risk factors for contralateral knee arthroplasty. In the multivariate model, only vertical angle of mechanical axis ≥3.03° (odds ratio [OR] 4.36, 95% confidence interval [CI], 2.47–9.11), K-L classification grades 3 and 4 (OR 2.46,3.72; 95%CI, 1.31–4.25, 1.98–6.87), and femoral tibial angle ≥187.32° (OR 6.32, 95%, 2.23–18.87) remained associated with the occurrence of knee arthroplasty.

About a quarter of patients with bilateral KOA received unilateral UKA will receive contralateral knee arthroplasty. Higher K-L classification, femoral tibial angle, and mechanical axis vertical angle are identified risk factors.

## 1 Introduction

Knee osteoarthritis (KOA) is a kind of degenerative disease of the knee joint, which is characterized by local articular cartilage destruction, accompanied by adjacent subchondral bone hyperplasia or lip-shaped bone.^[1]^ It is caused by mechanical, metabolic, inflammatory, and immune factors.^[2]^ Some studies have stated that approximately 13% of women and 10% of men older than 60 years suffering from symptomatic KOA.^[3,4]^ If not treated in time, it will cause joint deformity and dysfunction, and seriously affect the quality of life in patients. A multi-center, large-sample epidemiological survey has pointed out: the prevalence of KOA in middle-aged and elderly people over 40 years old is rising.^[5]^ Moreover, with the growing aging population and a desire for improved mobility and quality of life, these numbers are increasing rapidly.^[6]^ The main indication for knee arthroplasty is KOA, and it has been reported that patients with bilateral knee arthritis account for more than 2/3 of patients undergoing unilateral knee arthroplasty.^[7]^

Nowadays, unicompartmental knee arthroplasty (UKA) has been proven to be an effective treatment method for isolated medial compartment KOA in appropriately selected patients.^[8]^ Compared with total knee arthroplasty (TKA), UKA can preserve the anterior and posterior cruciate ligaments, and reduce the amount of osteotomy, these principles can preserve the common biomechanics of knee joint.^[9]^ Moreover, this surgical procedure shows the advantages of less intra-articular injury, fewer complications, and faster postoperative recovery of joint function. Nonetheless, fewer than 10% of all primary knee replacements are UKAs, even though up to half of all patients are potential UKA candidates.^[10]^

Traditionally, patients with severe advanced bilateral KOA often need bilateral knee arthroplasty. This surgical method can effectively eradicate the pain and greatly improve the quality of life of patients, which is highly advocated by the majority of orthopedists and more and more accepted by patients.^[11]^ Although some studies have confirmed that compared with single knee arthroplasty, double knee arthroplasty at the same time has the advantages of hospitalization cost, short recovery time, and less anesthesia acceptance. However, the study from Parisi et al^[12]^ showed that patients with bilateral KOA had improved symptoms of contralateral knee arthritis after unilateral arthroplasty. There have been a lot of studies on the prognosis of the contralateral knee after unilateral TKA and outcomes of the affected limb after receiving UKA.^[13]^ UKA offers a safe and efficient alternative to osteoarthritis, however, there are relatively few reports on the contralateral knee after UKA in patients with bilateral KOA. Currently, many studies have demonstrated the incidence and risk factors of contralateral TKA after primary TKA, however, few studies have reported the related data in regard to UKA.

Given that, this aim of our study was to demonstrate the incidence and risk factors of contralateral knee arthroplasty in patients who underwent primary unilateral UKA, so as to provide a theoretical basis for making a more comprehensive treatment strategy for patients with KOA. Moreover, based on the evidence, contralateral knee replacement could be a delay or avoid.

## 2 Materials and methods

This retrospective study was designed in accordance with the principles outlined in the Declaration of Helsinki. This study has been approved by the institutional review board of the Third Hospital of Hebei Medical University and all participants have signed informed consent forms.

### 2.1 Inclusion and exclusion criteria

Between June 2015 and December 2019, all patients who received unilateral UKA in the third department of joint orthopedics the third hospital of Hebei Medical University were recruited by querying electronic medical records (EMR). The inclusion criteria were as follows: (1) Participants suffered from bilateral KOA. (2) All participants received unilateral UKA for the first time. (3) Weight-bearing long X-ray film of both lower extremities were taken pre- and post-operative. (4) The clinical and imaging data of participants were complete. The exclusion criteria were as follows: (1) The patients had a history of knee arthroplasty. (2) Participants suffered from other lower limb orthopedic diseases or nervous respiratory or cardiac system diseases with limited function. (3) Participants suffered from severe postoperative complications including severe infection and fracture after primary surgery. (4) Participants suffered from rheumatoid arthritis. (5) Participants received revision surgery or underwent other surgery than arthroplasty for KOA during the follow-up period.

### 2.2 Operating method

All patients received routine intraoperative monitoring, such as oxygen saturation, electrocardiography, and non-invasive blood pressure measurements. All patients received spinal anesthesia, the operation was performed when the anesthesia plane was fixed at L2–3. The specific operations of the operation are as follows: (1) The affected lower limb was placed behind the special customized lower limb bracket, and the hip was bent for 30 minutes. At the same time, the leg sags naturally, which makes it flexion freely and the range of motion is more than 120°. The operation was performed after removing blood from the affected limb and applying a balloon tourniquet. (2) The medial parapatellar approach was taken to open the capsule of the knee joint to expose the medial compartment of the lesion. The integrity of the cartilage and anterior cruciate ligament in the weight-bearing area of the lateral compartment was examined and confirmed, and the proliferative bone tissue in the intercondylar fossa and the medial tibiofemoral space was removed. (3) Tibial osteotomy was performed by tibial extramedullary localization. The depth of tibial osteotomy was 2 to 3 mm below the deepest part of tibial erosion, which could accommodate the tibial test membrane and 4 mm thick liner. (4) The distal femoral condyle was removed under the guidance of a grinding bolt. (5) After the balance of flexion and extension space was measured, the prosthesis and pad were implanted. (6) Rinse the wound, electrocoagulation hemostasis, and knee flexion 45°. The incision was sutured in layers.

Postoperative management: (1) routine prevention of lower extremity deep venous thrombosis and infection after the operation.(2) After anesthesia, the patients were asked to do active quadriceps exercise and ankle pump exercise. (3) The knee bending function exercise was performed 24 hours after the operation and the walking exercise was gradually loaded.

### 2.3 Data collection

Two researchers (JCW and GY) inquired about patients’ EMR and made telephone follow-up to record patients’ demographics, surgery-related data, contralateral knee joint condition, and postoperative condition.

Demographic information of each participant such as age, body mass index (BMI), and gender were recorded carefully. Operation-related variables included surgical time, surgical side, surgeons’ experience, and interoperative blood loss. Radiological data: all patients were routinely taken weight-bearing long X-ray film of both lower extremities before the operation, the main measurement indexes included: (1) vertical angle of mechanical axis^[9]^; (2) internal and external joint space of knee joint; (3) Kellgren-Lawrence (K-L) classification of osteoarthritis; (4) femoral tibial angle^[10]^; and (5) hip knee ankle angle. The femoral tibial angle is the lateral angle between the femoral anatomic axis and the tibial anatomic axis.

The functional status of the knee and disease-related data including hospital for special surgery (HSS) score and visual analogous scale (VAS) were recorded to evaluate the function and pain of the knee.

### 2.4 Statistical analysis

All statistical analysis was performed with the Statistical Package for Social Sciences software (version 23.0; SPSS Inc., Chicago, IL, USA). The continuous data were expressed as mean ± standard deviation or median (interquartile range). First, a univariate logistic analysis was performed to evaluate the relationship between each categorical variable and contralateral knee arthroplasty. Whitney *U* test or *t* test was used to evaluate continuous variables when appropriate depending on the data distribution (equal variance and normality or not). Multivariate logistic regression analysis was used to evaluate the risk of subsequent knee arthroplasty on the non-operative side, dummy variables analysis was applied for K-L classification in the logistic analysis model to determine the relationship between this radiological indicator and contralateral knee arthroplasty. *P* values lower than .05 were interpreted as statistically significant in all the statistical analysis models.

## 3 Results

### 3.1 Characteristics of contralateral knee arthroplasty

**Figure 1:**
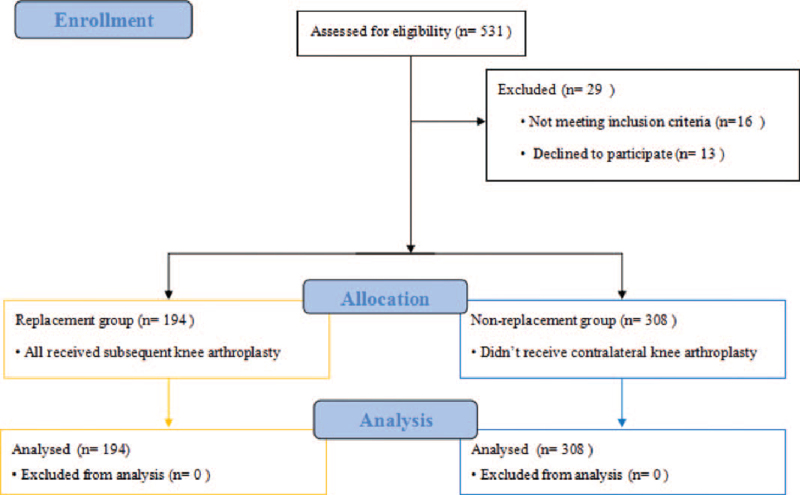
Flow diagram of study participants: replacement group (orange) and non-replacement group (blue).

Five hundred and thirty-one patients were assessed for study eligibility during this study. Sixteen patients did not meet the inclusion criteria and 13 patients declined to participate. Finally, a total of 502 patients were enrolled in this study. The Flow diagram of the study is shown in Figure 1. There were 201 males and 301 females, with a mean age of 61.2 ± 9.9 years (range 53–81 years). During the follow-up period, 194 patients receive subsequent knee arthroplasty and they are assigned to the replacement group. The other 308 patients did not receive contralateral knee arthroplasty and they were assigned to the non-replacement group. Hence, the incidence of contralateral knee arthroplasty is 38.64%. Table 1 shows the baseline characteristics of the study population. There were no significant differences in age, gender, BMI, surgical time, and surgical side between the 2 groups, although it seemed that patients were older (62.37 vs 60.25) and BMI was higher (28.34 vs 27.88) in the non-replacement group.

**Table 1:** Demographic and operative data of 2 groups of patients (χ ± *s*).

	Replacement group (n = 194)	Non-replacement group (n = 308)	*P* value
Age (yrs)	60.25 ± 9.37	62.37 ± 10.41	.234
BMI (kg/m^2^)	27.88 ± 3.252.97	28.34 ± 3.25	.338
Gender			.323
Female (%)	120 (61.86%)	181 (58.77%)	
Male (%)	74 (39.14%)	127 (41.23%)	
Surgical site			.547
Left (%)	91 (46.90%)	150 (48.70%)	
Right (%)	103 (53.10%)	158 (51.30%)	
Surgical time (min)	97.23 ± 21.37	100.25 ± 19.36	.281

Receiver operating characteristic analysis was performed to detect the optimum cutoff value for the femoral tibial angle and vertical angle of the mechanical axis which could be associated with the occurrence of contralateral knee arthroplasty. The statistical result shows that the critical value of femoral tibial angle and vertical angle of the mechanical axis were 187.32° (AUC = 0.795, sensitivity 83.2%, and specificity 90.1%) and 3.03° (AUC = 0.776, sensitivity 85.2%, and specificity 75.3%).

### 3.2 Univariate and multivariate logistic analysis

In the univariate analysis, vertical angle of mechanical axis ≥3.03°, knee joint's internal and external joint space, K-L classification of osteoarthritis, and femoral tibial angle ≥187.32° of the non-operating side were investigated to be the significant risk factors for contralateral knee arthroplasty. Other factors, including age, gender, BMI, surgical time, and surgical side were not associated factors with contralateral knee arthroplasty occurrence. The detailed information is presented in Table 2.

**Table 2:** Univariate analysis of factors associated with contralateral knee arthroplasty (χ ± *s*).

Risk factors	Replacement group (n = 194)	Non-replacement group (n = 308)	*P* value
Age (yrs)	60.25 ± 9.37	62.37 ± 10.41	.263
BMI (kg/m^2^)	27.88 ± 3.252.97	28.34 ± 3.25	.116
Gender			.297
Female (%)	120 (61.86%)	181 (58.77%)	
Male (%)	76 (39.18%)	123 (39.94%)	
Surgical site			.154
Left (%)	91 (46.90%)	150 (48.70%)	
Right (%)	103 (53.10%)	158 (51.30%)	
Surgical time (min)	97.23 ± 21.37	100.25 ± 19.36	.086
Vertical angle of mechanical axis of non-operating side (≥3.03°)	3.23 ± 1.06	2.34 ± 0.97	<.01
Internal joint space of non-operating side (mm)	23.25 ± 9.61	34.46 ± 10.33	.023
External joint space of non-operating side (mm)	69.76 ± 18.61	59.57 ± 16.34	.016
K-L classification of non-operating side 1/2/3/4	12/24/76/82	83/122/57/46	<.01
Femoral tibial angle of non-operating side (≥187.32°)	188.27 ± 9.63	180.34 ± 7.26	.006

In the multivariate model, vertical angle of mechanical axis ≥3.03°, K-L classification of osteoarthritis, knee joint's internal and external joint space, femoral tibial angle ≥187.32° of the non-operating side were risk factors with approximate significance (*P* < .1). After adjustment for confounding factors, vertical angle of mechanical axis ≥3.03°, and femoral tibial angle of the non-operating side were independent risk factors associated with the contralateral knee arthroplasty occurrence (*P* = .026, .008), and the adjusted OR was 4.36 (2.47–9.11) and 6.32 (2.23–18.87), respectively. K-L classification grade 1 was set as a reference, in the multivariate logistic analysis model K-L classification grades 3 or 4 were demonstrated to be a risk factor of the occurrence of contralateral knee arthroplasty (*P* = .013, .007), and the OR value were 2.46 (1.31–4.25) and 3.72 (1.98–6.87). The results of the Hosmer–Lemeshow test demonstrated adequate fitness (*X*^2^ = 5.832, *P* = .631). The detailed information is presented in Table 3.

**Table 3:** Multivariate logistic regression analysis of factors associated with contralateral knee arthroplasty.

	Odds ratio	95% CI	*P* value
Vertical angle of mechanical axis of non-operating side (≥3.03°)	4.36	2.47–9.11	.026
K-L classificationof non-operating side			
3	2.46	1.31–4.25	.013
4	3.72	1.98–6.87	.007
Femoral tibial angle of non-operating side (≥187.32°)	6.32	2.23–18.87	.008

Clinically, there were a number of statistically significant differences between the 2 groups in HSS score and VAS score of the contralateral knee before the operation. Preoperative HSS scores were significantly lower in the replacement group than in the non-replacement group (*t* = 3.687, *P* = .018). Preoperative VAS scores were significantly higher in the replacement group than in the non-replacement group (t = 3.921, *P* = .032) (Table 4).

**Table 4:** Preoperative VAS score and HSS score between 2 groups (χ ± s).

	Replacement group (n = 194)	Non-replacement group (n = 308)	*P* value
VAS score	4.35 ± 1.03	3.27 ± 0.97	.032
HSS score	60.34 ± 5.67	67.81 ± 5.49	.018

## 4 Discussion

KOA is a common joint disease in the elderly. At present, more and more patients are diagnosed with bilateral KOA. Therefore, necessary surgery should be applied as soon as possible to halt the progress of degeneration and improve the function of the knee joint. UKA is a successful surgical method commonly used for patients suffering from unicompartmental KOA. The aim of this study was to help clinicians analyze the risk factors of contralateral knee arthroplasty occurrence, so as to provide a more comprehensive diagnosis and treatment plan. In this study, we reviewed patients who suffered bilateral KOA but received unilateral UKA. Our investigation revealed that the incidence of contralateral knee arthroplasty was 38.64%. Preoperative contralateral knee mechanical axis vertical angle ≥3.03°, femoral tibial angle ≥187.32°, K-L classification 3 or 4 have a higher risk to develop contralateral knee arthroplasty.

In the previous studies, a lot of evidence have shown that gender and age are closely related to the occurrence and progression of osteoarthritis.^[14]^ Furthermore, the relationship between BMI and osteoarthritis has been confirmed in previous literature. Wolfe and Lane have shown that female patients with high body mass index are more likely to suffer from osteoarthritis.^[15]^ The increase of BMI will lead to the increase of knee joint pressure, which will lead to cartilage damage, osteophyte formation, and eventually joint destruction.^[16]^ However, in this study, the results showed age, gender, BMI, surgical time, and surgical side are not the risk factors of contralateral knee arthroplasty occurrence although it seemed that patients were older (62.37 vs 60.25) and BMI was higher (28.34 vs 27.88) in the non-replacement group. The potential reason maybe these patients with osteoarthritis risk factors pay more attention to the rehabilitation training of the affected limb, change the unhealthy living, and walking habits, thus reducing the incidence of contralateral joint replacement. Furthermore, all the enrolled patients were diagnosed with KOA and underwent primary knee arthroplasty, they all have been accompanied by advanced age, higher BMI, and many other risk factors. Therefore, those factors mentioned above would not be associated with subsequent contralateral knee arthroplasty.

K-L classification system is a grading method for the severity of KOA. According to the X-ray findings of the knee joint, it can be divided into 5 grades from light to heavy. The higher the K-L classification, the greater the degree of knee joint damage.^[17]^ The femoral tibial angle is the lateral angle between the femoral anatomical axis and the tibial anatomical axis. The femoral tibial angle of normal Asians is 176.0°–180.4° and the knee joint <176.0° is defined as gonycrotesis, knee joint >180.4° is defined as gonyectyposis. The lower limb mechanical axis, also known as the force axis, refers to the axis passing through the center of the hip joint, knee joint, and ankle joint. The vertical angle of the mechanical axis is the angle between the mechanical axis of the lower limb and the vertical line of the center of the human body. The vertical angle of the mechanical axis and femoral tibial angle are important indexes to reflect the degree of knee deformity.^[18]^ In this study, compared with the non-replacement group, there were statistically significant differences in mechanical axis vertical angle, medial and lateral joint space, femoral tibial angle, hip knee ankle angle, and K-L classification of the contralateral knee before operation in the replacement group. However, only mechanical axis vertical angle ≥3.03°, K-L classification 3 or 4 and femoral tibial angle ≥187.32° of the non-operating side were independent risk factors after multivariate analysis.

Mak et al showed that when the varus angle increases by 2°, the medial pressure of the knee joint will increase by 33%.^[19]^ In the load-bearing state, the medial side of the knee joint receives more pressure than the lateral side, which results in more severe wear of the medial articular cartilage than the lateral side, and then leads to unequal joint space between the medial and lateral sides of the knee joint. After unilateral UKA, due to trauma and pain, the patient did not dare to force the affected limb excessively. In the early stage of rehabilitation, the patients began to exercise their lower limbs. Their dependence on the non-operative side of the limb increases, and the structural and functional defects of the contralateral limb itself may lead to further aggravation of the non-operative side of the knee lesions, causing irreversible damage and increasing the risk of surgery. Some scholars have pointed out that after unilateral knee arthroplasty, the femoral tibial angle of the contralateral knee joint will increase in varying degrees.^[12]^ At the same time, the change of gait and the shift of body center of gravity after TKA increase the pressure of the contralateral knee, which leads to the acceleration of the degeneration of the contralateral knee and the aggravation of the symptoms of knee arthritis. Therefore, the risk of contralateral knee arthroplasty in patients with bilateral knee arthritis is greatly increased.^[20]^

HSS score is often used to evaluate the recovery of knee function after knee arthroplasty, which can comprehensively evaluate the movement of the patellofemoral joint and femoral tibial joint.^[21]^ VAS score is one of the most commonly used simple scales to evaluate patients’ subjective pain. Participants dont need to fill in complicated questionnaires, they just need to look at a “pain ruler” and say a number between 0 and 10. This method is simple, relatively objective, sensitive, and easy to be accepted by patients. As VAS and HSS are comprehensive evaluation indexes related to pain and knee function, they are more likely to be affected by various indicators. To reduce the impact of VAS and HSS on other indicators, We did not include VAS and HSS into the regression analysis. The results of this study showed that compared with the non-replacement group, the HSS score and VAS score of the replacement group were significantly different. Although the 2 indicators were not detected after the operation, the results still showed that the preoperative pain was also the reason for the patients to consider accepting the contralateral joint replacement again.

It is undeniable that there are still some limitations in this study. (1) This study is a retrospective study, there is a certain bias, may have a certain impact on the accuracy of the results. (2) In this study, only 1 to 5 years of postoperative follow-up cases were collected, and the follow-up time still needs to be extended to obtain more accurate results. (3) We only collected some research indexes of patients before operation, whether these indexes will change after the operation and the impact on the research results need to be further studied. (4) Some patients who have not been contacted by telephone follow-up may receive contralateral knee arthroplasty in another institution. We can not find the relevant data in their EMR, which may cause some interference in the final results of this study.

## 5 Conclusion

In summary, our data suggest if patients with bilateral KOA after receiving primary unilateral UKA, have a risk of progression to contralateral knee arthroplasty of 38.64%. With the increasing number of patients with KOA and increasing demand for UKA, Preoperative contralateral knee mechanical axis vertical angle, femoral tibial angle, K-L classification, HSS score, and VAS score can be used as important factors to recommend patients to receive reoperation, which provides a guarantee for patients with bilateral KOA to formulate a perfect diagnosis and treatment plan.

## Acknowledgments

The authors would like to thank all the members of the third department of joint orthopedics, the Third Hospital of Hebei Medical University for their great help and support.

## Author contributions

XDH designed the study and drafted the initial manuscript. HL and BCC reviewed and revised the manuscript. DCS and HYN coordinated and supervised data collection. JCW and GY collected and analyzed data.

**Conceptualization:** Xiaodan Huang.

**Data curation:** Decheng Shao, Haiyun Niu, Jianchao Wang.

**Formal analysis:** Jianchao Wang, Guang Yang.

**Methodology:** Xiaodan Huang.

**Resources:** Decheng Shao.

**Writing – original draft:** Xiaodan Huang.

**Writing – review & editing:** Hua Li, Baicheng Chen.
